# Language Barriers in Patients With Cardiovascular Disease: A Mixed Methods Study

**DOI:** 10.1016/j.jacadv.2022.100077

**Published:** 2022-09-21

**Authors:** Edward Wang, Tala Al-Rousan, Wali Mansour, Diego Oliva, Nicole Herrick, Birju Desai, Laith Alshawabkeh

In 2010, 21% of U.S. residents primarily spoke a language other than English compared with 10% in 1980.^1^ Limited English proficiency (LEP) has been shown to increase the risk of hospital readmission, poor understanding of medical situations and reporting of cardiovascular disease (CVD), and adverse medication reactions.^2^ As the population of patients with LEP grows, we must understand how LEP affects patients’ perceptions and engagement with health care providers.

In this mixed method study, we aimed to characterize the effect that language barriers have on patients with LEP. We used Q Methodology, an increasingly used approach in health and social sciences, to analyze human subjectivity about a specific issue.^3^ Inclusion criteria included any diagnosis of CVD, any non-English spoken language preference documented in the electronic health record, and age ≥18 years. The Institutional Review Board at the University of California, San Diego, approved this study.

Between June 2021 and March 2022, cardiology clinic schedules were prospectively reviewed for patients fulfilling the inclusion criteria. Eligible participants who provided informed consent were enrolled during their clinic encounter. The Q-sort exercise comprised 23 statements developed from Terui’s framework for how language barriers create health disparities and from unstructured in-person interviews with key informants.^4^ The Q-sort exercise was administered in Spanish and Arabic after being translated from English using a certified clinical translation agency. Using an online Q Methodology tool, participants organized the 23 statements into a quasi-normal grid based on their level of agreement with each statement.^5^ Pearson correlation and centroid extraction with varimax rotation were used to extract 3 distinct factor groups. Participants were loaded into a factor group if they had a significant factor loading (*P* < 0.05) for only 1 group. Those who did not fulfill this criterion were iteratively assigned to the group that most closely reflected their responses. As a result, each factor group comprised participants who ranked the statements in a similar order and therefore represented a unique perspective about LEP barriers. We then analyzed the demographic characteristics and qualitatively evaluated the unique ordering of statements for each factor group.

Forty-six participants were enrolled, with 18, 19, and 9 belonging to groups 1, 2, and 3, respectively. The cumulative explained variance was 48.5%. Factor ranks for each group and statement are shown in Table 1. The mean age was 62 ± 15 years, and 61% were female. Spanish was the primary spoken language in 96%, and the remainder spoke Arabic. Forty-one percent did not speak any English, and 59% spoke English less than “well.” The average time spent in the United States was 31 ± 16 years, and 83% completed high school or less. These demographic characteristics did not vary significantly among the 3 groups. Hypertension and congestive heart failure were the most common CVD, and there were no significant differences in the types or distribution of CVD among the 3 groups.

**Table 1:** Factor Ranks for Each Q-Sort Statement and Their Associated Significance for Each Factor Group

Statement	Group 1	Group 2	Group 3
1. I feel comfortable using English in routine daily activities.	0	1	−2
2. I feel comfortable using English in healthcare settings.	−1	1	−1
3. The language barrier has made it difficult for me to access healthcare.	−1	0	−2
**4. Because of the language barrier, I explicitly seek out providers who speak my language.**	**0**	**2**	**0**
5. I have had delays in care because of my language barrier.	−1	0	−3
6. The language barrier has made me hesitant to ask all my questions during appointments.	1	−1	0
7. The language barrier makes it difficult to have all my questions answered during appointments.	0	0	1
8. I feel guilty about my language barrier being a burden on the healthcare system and providers.	−2	0	1
**9. Because of the language barrier, I look for and use additional tools and methods to improve communication between my providers and me, such as bringing family to translate for me.**	**2**	**1**	**0**
10. My doctor’s interpreter service is helpful and adequate to accurately translate all of my concerns.	2	3	1
11. A video interpreter service is as good as an in-person professional interpreter.	1	1	−1
12. I would rather use my own interpreter than use an interpreter provided by the clinic or hospital.	3	−1	−1
13. I have had healthcare appointments cut short because of the language barrier.	0	−3	0
14. My appointments should be booked for longer time slots because of the language barrier.	1	−2	−1
15. I have experienced discrimination in healthcare settings on the basis of my language.	−3	−2	−2
16. I have experienced discrimination in healthcare settings on the basis of my race or ethnicity.	−3	−2	−3
17. I have been dismissed, or I have had my concerns/questions dismissed in healthcare settings because of my language preference.	−2	−1	0
18. I am satisfied with the accuracy and availability of translated educational resources for my heart disease, besides my own healthcare providers, such as websites and brochures.	1	3	3
**19. Because of my language barrier, I feel isolated and am unable to communicate with those around me (including friends and family) about my heart disease.**	**−2**	**−1**	**2**
**20. Because of the language barrier, other non-medical people who are involved in my care (such as friends and family) do not have a good understanding of my own disease.**	**−1**	**0**	**2**
21. The language barrier has made it difficult for me to understand the causes, complications, and management of my own heart disease.	0	−3	3
22. I feel that the quality of my healthcare is similar to those who speak English.	3	2	2
23. I feel that my own health and well-being are similar to those who speak English.	2	2	1

Factor ranks and colors denote the level of agreement with each statement (−3/red = strongly disagree; 0/white = neutral; 3/green = strongly agree). Distinguishing statements are **bolded** and can be used in clinical settings to identify patients in each factor group.

Group 1 comprised empowered participants in successful therapeutic alliances with the health care system and were uniquely characterized by their independent search for resources outside of their providers to improve health literacy. Although they did not experience discrimination, dismissal, or guilt from speaking a different language, they were hesitant about asking questions during appointments due to perceived time constraints. They also preferred to use friends or family to interpret. They also successfully shared their health care experiences with those around them and felt that their health and health care were equivalent to English-proficient patients.

Group 2 was similar to Group 1, except its members uniquely and explicitly sought language-concordant providers, which sometimes led to delays in care and increased guilt about being a burden on health care systems. Compared with Group 1, they were more willing to use system-provided resources and interpreters because these services adequately addressed their needs. Similarly, the language barrier did not negatively impact their health or health care experiences through discrimination or isolation.

Finally, Group 3 comprised isolated individuals in an unsupportive environment. They were more likely to live with an English speaker at home than Group 1, felt more guilt and dismissal, and were less likely to be satisfied with interpretation services. Most characteristically, they experienced health care in isolation because they could not communicate with those around them about their disease. Consequently, they felt that their health was worse than English-proficient patients.

Our study highlights several areas for future research and intervention. First, providers should attempt to identify the group to which their patients belong. If they belong to Group 1 or 2, providers should increase access to outside resources or language-concordant services to address their health care needs. Second, the unique isolated experiences of Group 3 patients necessitate further investigation into the effect of social networks on health disparities. Providers might consider organizing support groups to address this need. Finally, future interventions should target practices that create unequal experiences, such as the guilt and poorly perceived health distinctively reported by Group 3 patients. Reducing appointment time pressures that disproportionately affect patients with LEP is especially important. This study is among the first to analyze in-depth data from LEP patients with CVD using mixed methods. However, limited generalizability to patients in other geographical locations is a limitation. Our study highlights several specific areas for targeted interventions to ensure equitable health outcomes and experiences for the understudied population of CVD patients with LEP.
